# Role of Intestinal LXRα in Regulating Post-prandial Lipid Excursion and Diet-Induced Hypercholesterolemia and Hepatic Lipid Accumulation

**DOI:** 10.3389/fphys.2017.00280

**Published:** 2017-05-09

**Authors:** Tibiábin Benítez-Santana, Sarah E. Hugo, Amnon Schlegel

**Affiliations:** ^1^University of Utah Molecular Medicine Program, School of Medicine, University of UtahSalt Lake City, UT, USA; ^2^Division of Endocrinology, Metabolism and Diabetes, Department of Internal Medicine, School of Medicine, University of UtahSalt Lake City, UT, USA; ^3^Department of Biochemistry, School of Medicine, University of UtahSalt Lake City, UT, USA; ^4^Department of Nutrition and Integrative Physiology, College of Health, University of UtahSalt Lake City, UT, USA

**Keywords:** Liver X Receptor, intestine, postprandial lipemia, triglycerides, cholesterol, chylomicrons, zebrafish

## Abstract

Post-prandial hyperlipidemia has emerged as a cardiovascular risk factor with limited therapeutic options. The Liver X receptors (Lxrs) are nuclear hormone receptors that regulate cholesterol elimination. Knowledge of their role in regulating the absorption and handling of dietary fats is incomplete. The purpose of this study was to determine the role of intestinal Lxrα in post-prandial intestinal lipid transport. Using Lxrα knockout (*nr1h3*^−/−^) and intestine-limited Lxrα over-expressing [*Tg*(*fabp2a:EGFP-nr1h3*)] zebrafish strains, we measured post-prandial lipid excursion with live imaging in larvae and physiological methods in adults. We also conducted a long-term high-cholesterol dietary challenge in adults to examine the chronic effect of modulating *nr1h3* gene dose on the development of hypercholesterolemia and hepatic lipid accumulation. Over-expression of Lxrα in the intestine delays the transport of ingested lipids in larvae, while deletion of Lxrα increases the rate of lipid transport. Pre-treating wildtype larvae with the liver-sparing Lxr agonist hyodeoxycholic acid also delayed the rate of intestinal lipid transport in larvae. In adult males, deletion of Lxrα accelerates intestinal transport of ingested lipids. Adult females showed higher plasma Lipoprotein lipase (Lpl) activity compared to males, and lower post-gavage blood triacylglycerol (TAG) excursion. Despite the sexually dimorphic effect on acute intestinal lipid handling, *Tg*(*fabp2a:EGFP-nr1h3*) adults of both sexes are protected from high cholesterol diet (HCD)-induced hepatic lipid accumulation, while *nr1h3*^−/−^ mutants are sensitive to the effects of HCD challenge. These data indicate that intestinal Lxr activity dampens the pace of intestinal lipid transport cell-autonomously. Selective activation of intestinal Lxrα holds therapeutic promise.

## Introduction

Atherosclerosis remains the leading cause of death (Lozano et al., 2012). The major driver of atherosclerosis is increased circulating cholesterol-rich lipoprotein particles (Stamler et al., 1986). Statin drugs, which decrease hepatic cholesterol synthesis and thereby promote hepatic cholesterol-rich lipoprotein particle clearance, are potent reducers of the risk of death from atherosclerosis (Cholesterol Treatment Trialists' (CTT) Collaboration et al., 2015). Nevertheless, these drugs do not fully ameliorate the risk of ischemic cardiovascular events, ultimately reflecting gaps in understanding of the drivers of atherosclerotic progression.

The persistence of TAG-rich lipoproteins in the post-prandial state has emerged as a major risk factor for atherosclerosis (Jørgensen et al., 2014; The TG and HDL Working Group of the Exome Sequencing Project et al., 2014). Namely, in large prospective cohort studies, risk of atherosclerosis was found to be attributable to the non-fasting circulating TAG levels, which are a proxy measurement of atherogenic lipoprotein particles (chylomicron remnants, intermediary density lipoprotein, IDL; and small, dense low density lipoprotein particles) that should normally not accumulate in the circulation. Indeed, this population-level investigation has pointed to hereditable defects in vascular clearance of TAG-rich lipoproteins, and driven drug development for Mendelian causes of severely elevated TAG levels (Gaudet et al., 2014). For the general population, there are no effective therapies available to blunt post-prandial hypertriglyceridemia (Nordestgaard and Varbo, 2014).

A better understanding of the molecular cues governing intestinal fat absorption, storage and lipoprotein production may lead to new therapies to decrease post-prandial hyperlipidemia (Dash et al., 2015; Giammanco et al., 2015). A detailed gene-regulatory scheme governing intestinal lipid physiology is still lacking, although much progress has been made in defining the machinery of lipid absorption and lipoprotein production (Iqbal and Hussain, 2009; Abumrad and Davidson, 2012; Dash et al., 2015). The most apparent gaps in our understand of intestinal lipid handling relate to how and why enterocytes store a substantial fraction of absorbed lipids in cytoplasmic lipid droplets, and the cues that control this storage and subsequent release (Robertson et al., 2003; Zhu et al., 2009; Douglass et al., 2012; Dash et al., 2015; Giammanco et al., 2015).

Lxrs are nuclear hormone receptors whose major endogenous ligands are oxysterols, cholesterol catabolites that accumulate in proportion to cholesterol excess (Janowski et al., 1999), and select sterane intermediates of cholesterol synthesis and bile acids (Song et al., 2000; Yang et al., 2006). When activated by ligands, Lxrs drive a multi-organ gene transcriptional program that induces cholesterol elimination (Calkin and Tontonoz, 2012). Genetic activation of Lxrα in the intestine blunts cholesterol absorption (Lo Sasso et al., 2010) and fatty acid absorption (Cruz-Garcia and Schlegel, 2014); and drives the absorbed lipids into a cytoplasmic lipid droplet pool (Cruz-Garcia and Schlegel, 2014). Pharmacological activation of intestinal Lxrs also blunts fatty acid absorption and transport (Briand et al., 2016). Conversely, global deletion of mouse Lxrα increases the fraction of dietary cholesterol that is absorbed in the presence of a non-sterol Lxr agonist, an effect not seen when Lxrβ is deleted (Hu et al., 2012). Since oxysterols are excreted in bile (Mutemberezi et al., 2016), these studies argue that the intestine is exposed to multiple Lxr-activating signals that could dampen lipid transport with every meal. Unfortunately, Lxrα activation with synthetic non-sterol ligands up-regulates hepatic lipogenesis and Very Low Density Lipoprotein (VLDL) particle secretion (Repa et al., 2000; Schultz et al., 2000; Grefhorst et al., 2002; Bradley et al., 2007). This property has impeded Lxr-based drug development (Kirchgessner et al., 2016).

Zebrafish models of dyslipidemia have emerged in recent years as a powerful system for studying intestinal lipid transport, vascular lipoprotein metabolism, and the early steps of atherosclerosis (Fang et al., 2014; Schlegel, 2016). Most notably, zebrafish have similar abundance and distribution of circulating lipoproteins as humans (Stoletov et al., 2009; Liu et al., 2015), unlike numerous preclinical models (Yin et al., 2012). Additionally, examination of intestinal lipid handling has been studied extensively in zebrafish with results concordant with findings seen in Mendelian Diseases (Schlegel and Stainier, 2006; Avraham-Davidi et al., 2012; Levic et al., 2015).

It is important to stress that zebrafish carry a singly Lxrα ortholog, whose encoding gene is syntenic to human LXRα (Reschly et al., 2008; Cruz-Garcia et al., 2009; Fonseca et al., 2017). The Lxrβ locus was lost in the fish phylum (Fonseca et al., 2017). Furthermore, zebrafish Lxrα binds and is activated by endogenous and synthetic ligands in a manner similar to human LXRα (Archer et al., 2008; Reschly et al., 2008). Finally, human intestines express LXRα only (Uhlén et al., 2015). In this study we examine the effect of modulating zebrafish intestinal Lxrα activation on intestinal lipid transport and on the accumulation of vascular and hepatic lipids using new live imaging and physiological methods on our previously described Lxrα deletion and intestinal over-expression lines. We show intestinal Lxrα activation delays transport of ingested lipids, decreasing the post-prandial plasma TAG excursion. This effect protects animals from HCD-induced hepatic lipid accumulation. The methods we have optimized will be of broad use to others interested in studying aspects of enterocyte handling of lipids.

## Methods

### Animals

The Institutional Animal Care and Use Committee of the University of Utah approved all studies. Animals were euthanized by tricaine overdose or immersion in ice. The Centralized Zebrafish Animal Resource (CZAR) at the University of Utah maintains the wildtype (WT) WIK strain zebrafish used in this study. The *nr1h3*^*z101a*^, and *Tg*(*fabp2a:EGFP-nr1h3*)^*z103*^ lines were described previously (Cruz-Garcia and Schlegel, 2014). The *nr1h3*^*z101a*^ mutant strain was targeted with Transcriptional Activator-like Effector Nucleases to create an in-frame stop mutation within the DNA binding domain (i.e., it is a null mutation). The *Tg*(*fabp2a:EGFP-nr1h3*)^*z103*^ line drives Lxrα expression in enterocytes, and animals carrying a single-copy of the transgene (heterozygous) were used in all experiments.

### Larval gavage and whole-mount epifluorescence microscopy

Four nanoliters of a 5:1 mixture (*v:*v) of triolein and cholesteryl oleate containing 1:1000 (*v*:*v*) dilution of cholesteryl BODIPY® 542/563 undecanoate (CE_11_-BODIPY) was gavaged into the proximal intestines of zebrafish larvae exactly as described (Cocchiaro and Rawls, 2013). After gavage, and between live microscopic scoring sessions, animals were maintained at 28°C, with a 14-h light:10-h dark cycle. Red fluorescent signal in the vasculature was monitored with a Leica M 205 FA stereomicroscope fitted with a camera and computer in a blinded fashion. Six larvae from each genotype were studied simultaneous, and the results are presented as the mean ± standard error of the mean (*n* = 18 larvae in total per each analysis). For experiments involving hyodeoxycholic acid (HDCA, Sigma), the compound was dissolved in dimethylsulfoxide, and 4 μL of vehicle or of a 5 μM solution was gavaged 24 h prior to gavage of the lipid mixture.

### Tyloxapol injection, oral lipid gavage, lipoprotein lipase activity

Three months post-fertilization (mpf) adult zebrafish were injected intraperitoneally with 2.5 mg/g tyloxapol (Millar et al., 2005). Animals were then subjected to an oral gavage with 0.1 mL of a 5:1 (*v*:*v*) mixture of olive oil and cholesteryl ester. In pilot experiments, plasma TAG peaked 8 h after oral gavage. Thus, 5 and 8 h after oral gavage, animals underwent terminal phlebotomy and plasma TAG was measured. Plasma Lpl activity was measured using a commercial kit (Cayman Chemicals, Michigan, USA) exactly as described on plasma collected at the 8-h time point to confirm suppression of Lpl activity throughout the experimental window (Liu et al., 2015). Lpl activity was measured in a 15-min *ex vivo* assay, and the slope of each activity curve was taken as the rate constant for the Lpl activity present.

### High cholesterol feeding

The high cholesterol (4% *w*/*w*) diet was prepared as previously described (Stoletov et al., 2009). Commercial flakes (TetraMin Tropical Flakes, Blacksburg, VA) were soaked in a cholesterol-diethyl ether solution and the flakes were left to dry overnight. For all adult studies, 5 female and 5 male 3 mpf zebrafish were distributed in 3L tanks. The animals were fed twice daily with automatic feeders for 7 months. Never-mated females were housed separately to avoid confounding issues of post-spawning vitellogenesis marked by transient hepatic steatosis (Sheridan, 1988; Babin and Vernier, 1989).

### Blood and tissue lipid composition analysis

Blood was collected by cardiac puncture with heparinized glass capillaries attached to a microinjector (Microinjection Systems, Harvard System). Blood was diluted in 0.2 mL tubes with 20 μL of PBS-EDTA. After centrifugation at maximum speed for 5 min, plasma was collected. Tissues were homogenized in lysis buffer (20 mM Tris-HCl, 150 mM NaCl, 1 mM EDTA, 1 mM EGTA, 1% Triton X-100) by sonication. Protein concentration was determined with the BCA protein assay reagent (Thermo Scientific). The TAG and total cholesterol levels in the blood were analyzed with colorimetric assay kits (Spinreact, Mexico).

Total lipids were Folch-extracted from liver (Iverson et al., 2001). Unesterified cholesterol, cholesteryl esters, TAG, and free fatty acids were resoled using thin layer chromatography exactly as we described previously (Schlegel and Stainier, 2006; Hugo et al., 2012; Karanth et al., 2013). The abundance of each lipid class was quantified using a standard charring and copper-based densitometric assay (Bitman and Wood, 1982; Ruiz and Ochoa, 1997), with normalization of lipid abundance to protein content (Cruz-Garcia and Schlegel, 2014).

### RT-PCR

RNA was extracted from liver and analyzed for *acaca, fasn, hmgcra, srebf1*, and *srebf2* abundance exactly as we described previously, using the *rpp0* transcript for normalization (Karanth et al., 2013; Cruz-Garcia and Schlegel, 2014). Intestinal *abca1a* and *abca1b* abundance was quantified from intestines using 5′-CCACATCGAGGACTACTCCG and 5′-TGTCTCTTTGGCCTTCTCGT; and 5′-TCTCCCAGACCACACTAGACC and 5′-TTTGGTCCTTCGCAAAGTTT, respectively.

### Statistical analysis

Statistical analyses were performed using DataGraph 4.1 (Visual Data Tools) and SPSS 19.0 (IBM) software. Data are presented as means ± standard error of the mean. The normality of the variable distribution was verified using Levene's test; and the data did not require transformation. Unless explicitly stated otherwise, differences with the WT group were evaluated using Student's *t*-test or 1-way ANOVA, with indicated parametric tests. A significance of *P* < 0.05 was applied to all statistical tests performed.

## Results

### Lxrα gene dose regulates the rate of transport of gavaged lipids in zebrafish larvae

**Figure 1:**
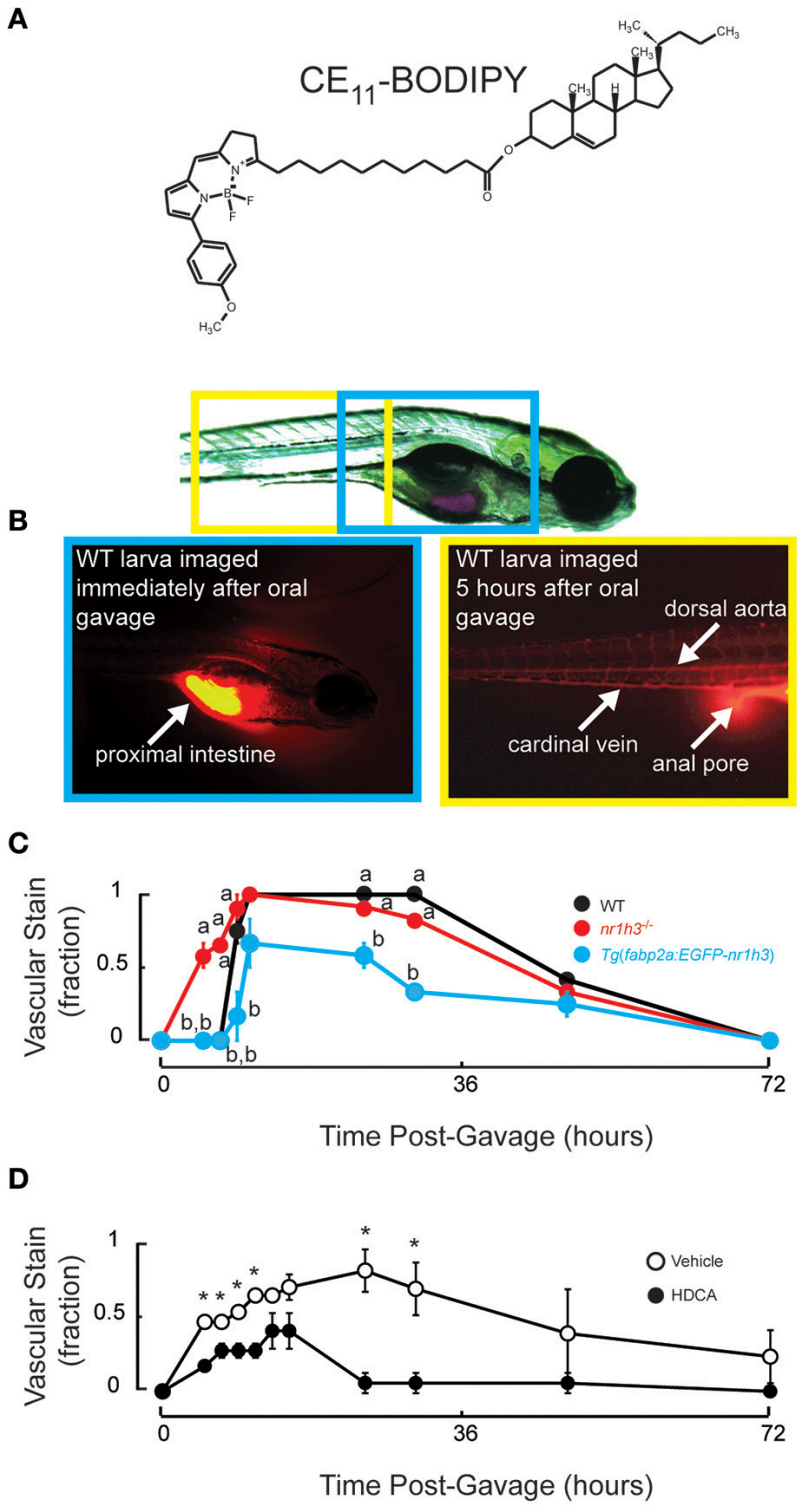
**Genetic activation of intestinal Lxrα regulates the pace of transport of ingested lipids in larvae. (A)** Structure of CE_11_-BODIPY. **(B)** A 7 days post-fertilization (dpf) larva following oral gavage of a mixture of triolein, cholesteryl oleate and CE_11_-BODIPY (upper). Immediately after oral gavage, the fluorescent signal is very strong in the anterior intestine (lower left). Within 5 h of gavage, strong vascular staining is apparent, and excess (not absorbed) label can be seen passing through the anal pore (lower right). **(C)** Six dpf WT, *nr1h3*^−/−^, and *Tg*(*fabp2a:EGFP-nr1h3*) transgenic larvae underwent gavage with the lipid mixture, and were scored for the appearance of lipids in the vasculature in a blinded fashion. Values not sharing a common superscript letter are significantly different at *P* < 0.05 at each time point in 2-sided student *t*-tests vs. WT; *n* = 18 for each genotype. **(D)** Five dpf WT larvae underwent gavage with vehicle or HDCA. Twenty-four hours later larvae were gavaged with the lipid mixture, and were scored for the appearance of lipids in the vasculature in a blinded fashion. ^*^P < 0.05 at each time point in 2-sided student *t*-tests vs. WT; *n* = 18 for each genotype.

Intestinal Lxrα over-expression induces a gene expression program that diverts absorbed lipids into a cytoplasmic lipid droplet pool (Cruz-Garcia and Schlegel, 2014); however, whole-mount Oil Red O (ORO) histological staining proved insensitive in revealing differences in vascular lipid accumulation between WT and *nr1h3*^−/−^ larvae (Cruz-Garcia and Schlegel, 2014). Thus, we performed oral gavage to deliver a defined bolus of neutral lipids and the fluorescent lipid CE_11_- BODIPY, whose fatty acyl chain can be incorporated into neutral and phospholipids *in vivo* (Carten et al., 2011), into the proximal intestine of zebrafish larvae (Figure 1A). Immediately after gavage, fluorescent signal was seen only in the lumen of the intestine, and starting at 5 h post-gavage, fluorescent signal could be seen in the vasculature (Figure 1B). After gavage, *nr1h3*^−/−^ mutant larvae showed more rapid vascular lipid accumulation compared to WT and *Tg*(*fabp2a:EGFP-nr1h3*) larvae (Figure 1C). All WT and *nr1h3*^−/−^ mutant larvae showed vascular fluorescent lipid accumulation by 10.5 h after oral gavage, while approximately one-third of *Tg*(*fabp2a:EGFP-nr1h3*) transgenic larvae had no vascular lipid accumulation. These results, obtained by repeated imaging of live larvae, confirm and extend our previous findings with fixed larvae, and revealed a difference between WT and *nr1h3*^−/−^ mutants that was not apparent when examining animals fed a lipid rich meal and then fixed and stained with ORO (Cruz-Garcia and Schlegel, 2014).

We previously found using a less sensitive ORO-based staining method that *nr1h3*^−/−^; *Tg*(*fabp2a:EGFP-nr1h3*) larvae showed delayed appearance of lipids in the vasculature following a fatty meal (Cruz-Garcia and Schlegel, 2014). This finding suggested that enterocyte over-expressed Lxrα acts cell autonomously to regulate the pace of chylomicron appearance. This genetic finding and the results in Figure 1C, nevertheless, raise the possibility that life-long changes in gene expression in other tissues might contribute to the pace of dietary lipid transport. To address this issue, we subjected WT larvae to oral gavage with the liver-sparing (i.e., does not induce de novo lipogenesis) Lxrα agonist HDCA (Singhal et al., 1984; Cohen-Solal et al., 1995; Song et al., 2000; Sehayek et al., 2001; Shih et al., 2013; De Marino et al., 2017). Twenty-four hours later, we performed a second oral gavage of the fluorescently labeled lipid mixture. Figure 1D shows that compared to pre-gavage with vehicle, HDCA pre-gavage delayed the appearance of fluorescent lipid in the vasculature and blunted the peak fraction of animals with vascular lipid staining. Thus, acute intestinal Lxrα activation is sufficient to blunt transport of absorbed lipids.

### Adult zebrafish have sexually dimorphic plasma Lpl activity

**Figure 2:**
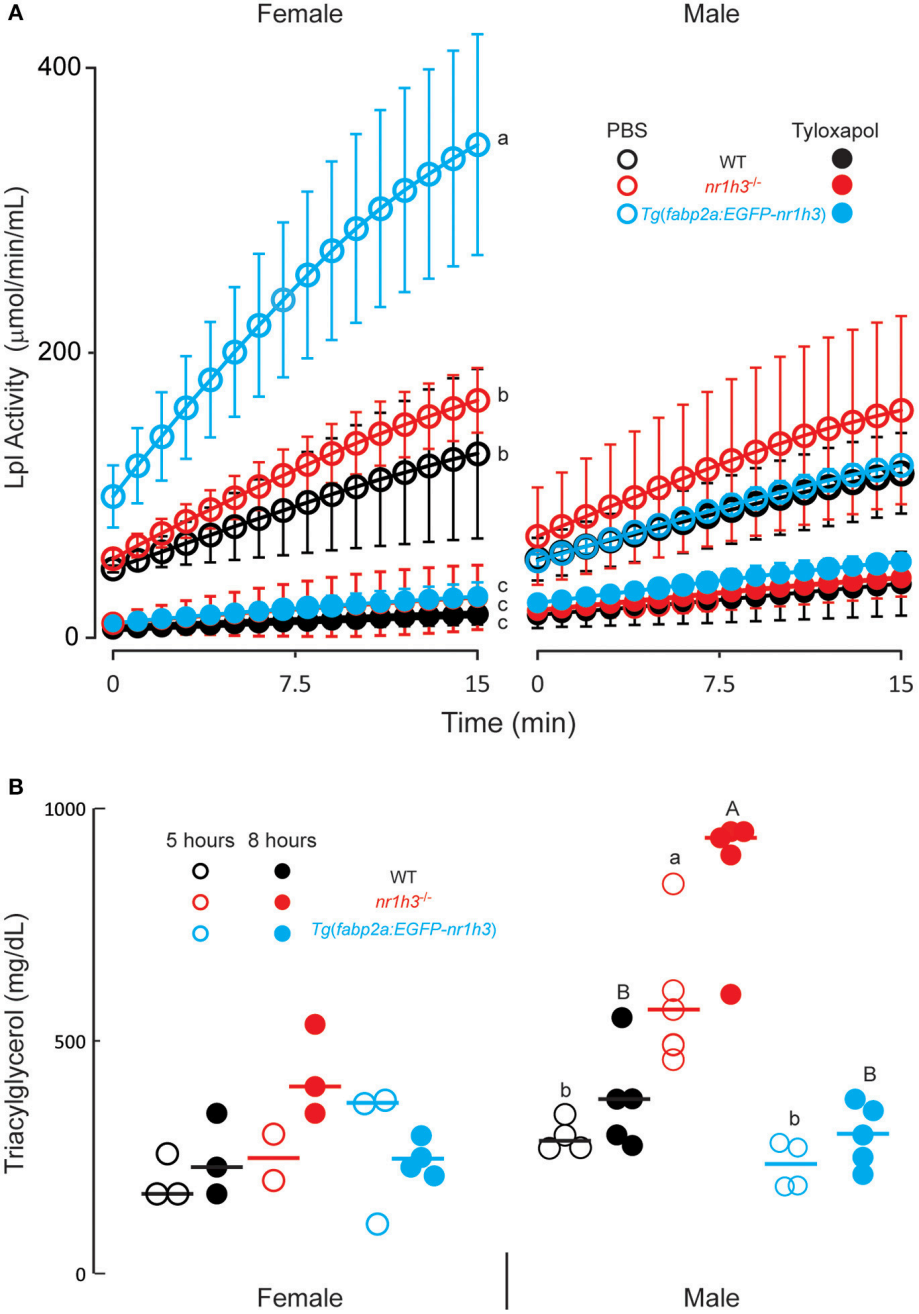
**Genetic activation of intestinal Lxrα regulates the pace of transport of ingested lipids in adults. (A)** Lipoprotein lipase (Lpl) activity 8 h after intraperitoneal injection of PBS or Tyloxapol (*n* = 6 for each line). **(B)** Plasma TAG concentration at the indicated times following oral gavage of lipids. Values not sharing a common superscript letter are significantly different at *P* < 0.05 in 1-way ANOVA.

To test whether the differences among the three cohorts of larval persist into adulthood, we measured post-gavage TAG in animals injected intraperitoneally with the Lpl inhibitor tyloxapol (Millar et al., 2005) at a concentration that inhibited serum Lpl activity for the duration of the experimental window. This standard approach avoids the potential contribution of differential lipolysis to the measured TAG. When injected with PBS, we found that females showed higher plasma Lpl activity in this *ex vivo* assay compared to males, with *Tg*(*fabp2a:EGFP-nr1h3*) transgenic females showing the highest activity (Figure 2A). Tyloxapol successfully inhibited Lpl activity in both sexes and among all genotypes; however, the decreases in Lpl activity following tyloxapol injection were only significantly lower in females (Figure 2A).

### Adult male zebrafish have greater post-gavage lipid excursion, which is modulated by Lxrα gene dose

After confirming complete inhibition of Lpl over the course of the experimental window, we measured post-gavage plasma TAG excursions. Male *nr1h*3^−/−^ animals showed the greatest increase in post-gavage serum TAG concentrations, at both time points examined (Figure 2B). Both *Tg*(*fabp2a:EGFP-nr1h3*) males and females showed decreased post-gavage serum TAG at both 5 and 8 h after gavage, although the difference was only significant in males compared to *nr1h3*^−/−^ animals. These results, while revealing a previously unreported sexual dimorphism in zebrafish plasma Lpl activity, recapitulate the larval phenotypes we observed.

### Adult zebrafish lacking Lxrα show severe hypercholesterolemia after HCD challenge

**Figure 3:**
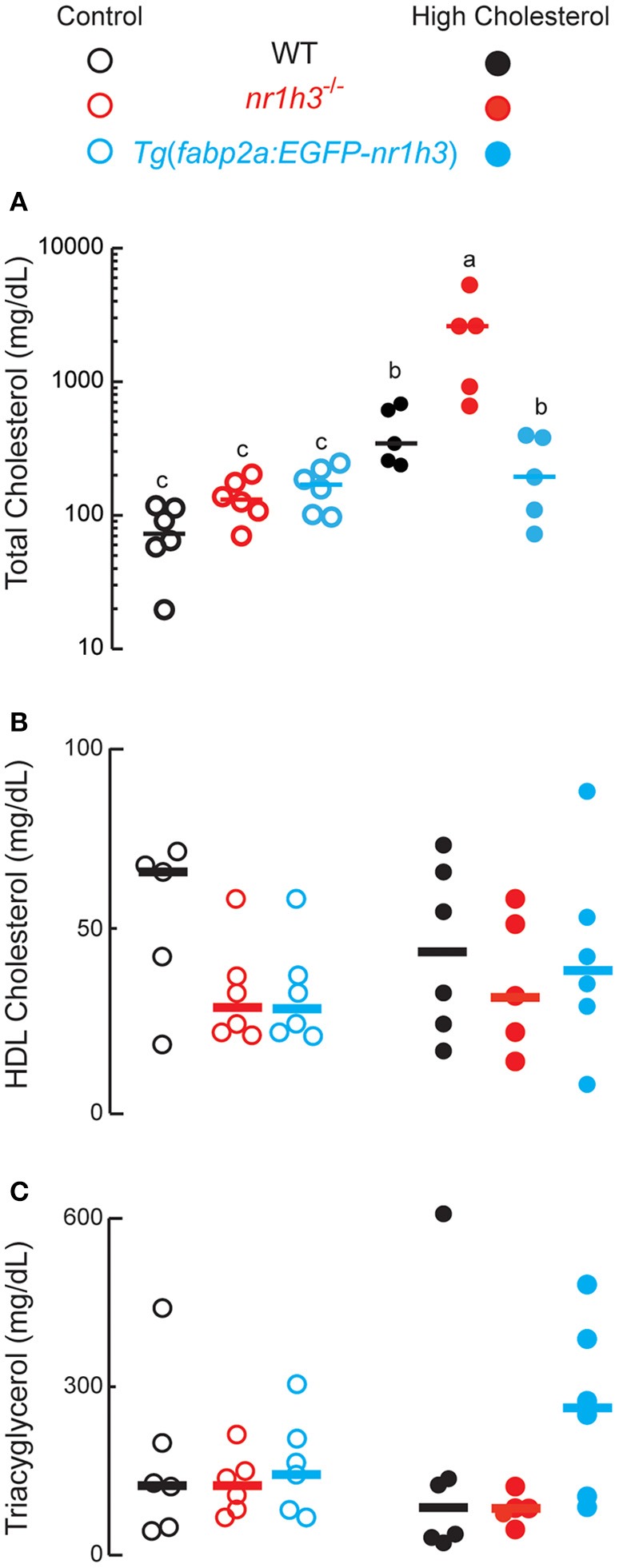
**Genetic activation of intestinal Lxrα protects against high-cholesterol diet-induced hypercholesterolemia. (A–C)**, WT, *nr1h3*^−/−^, and *Tg*(*fabp2a: EGFP- nr1h3*) transgenic animals were fed control diets and HCD from 3 to 10 months post-fertilization. The *nr1h3*^−/−^ animals showed a statistically significant increase in plasma total cholesterol on the high cholesterol diet, while *Tg*(*fabp2a:EGFP-nr1h3*) transgenic animals had lower (but still increased compared to control-diet fed animals) total cholesterol than WT animals. Equal numbers of males and females were used in each cohort. The median values are shown with a horizontal line. Values not sharing a common superscript letter are significantly different at *P* < 0.05 in 1-way ANOVA.

Next, we conducted a long HCD-feeding experiment to examine the effect of modulating intestinal Lxrα gene dose on the development of dyslipidemia and hepatic lipid accumulation. The *nr1h3*^−/−^ animals developed severe hypercholesterolemia, with a median value over 2,000 mg/dL at the conclusion of the HCD feeding period (Figure 3A). WT and *Tg*(*fabp2a:EGFP-nr1h3*) animals were protected from hypercholesterolemia to a similar degree. The plasma HDL cholesterol (Figure 3B) and TAG (Figure 3C) were not significantly different among the three genotypes on either diet.

### Adult zebrafish lacking Lxrα show severe hepatic lipid accumulation, while animals over-expressing Lxrα in the intestine are protected from hepatic lipid accumulation

**Figure 4:**
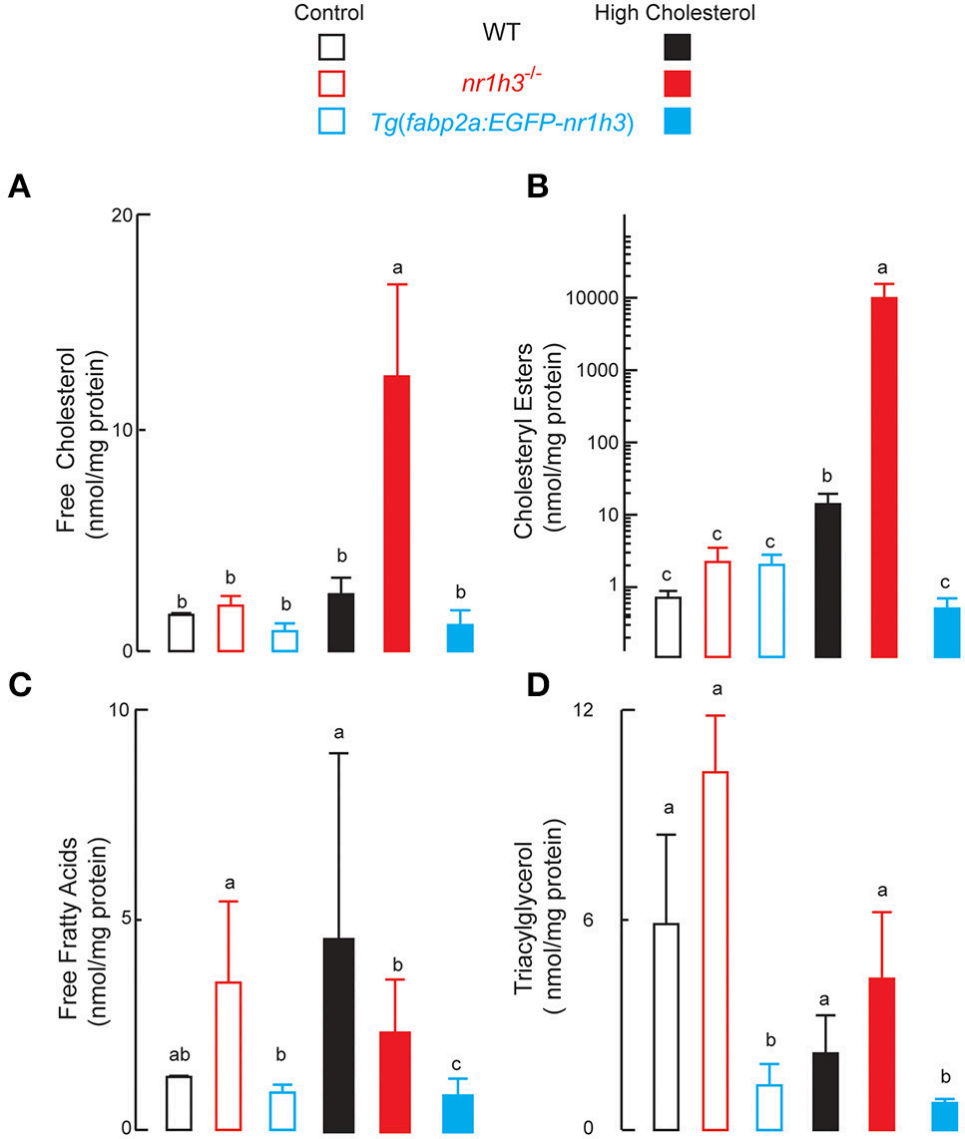
**Genetic activation of intestinal Lxrα protects against high-cholesterol diet-induced hepatic lipid accumulation. (A–D)**, livers were harvested following phlebotomy (in Figure 3). Lipids were extracted and analyzed with thin layer chromatography. Equal numbers of males and females were used in each cohort (*n* = 10). Values not sharing a common superscript letter are significantly different at *P* < 0.05 in 1-way ANOVA.

The livers of *nr1h3*^−/−^ animals had significant increases in free and esterified cholesterol concentrations under HCD feeding, while *Tg*(*fabp2a:EGFP-nr1h3*) livers had significantly lower values (Figures 4A,B). Hepatic free fatty acids were significantly lower in *Tg*(*fabp2a:EGFP-nr1h3*) animals; however, diet did not affect this difference (Figure 4C). Liver TAG were significantly lower in *Tg*(*fabp2a:EGFP-nr1h3*) livers compared to *nr1h3*^−/−^ animals, and there were significant differences between dietary cohorts, with all three genotypes showing lower liver TAG under HCD (Figure 4D). These fasting blood and liver lipid parameters were not sexually dimorphic, and are shown in aggregate for both sexes.

**Figure 5:**
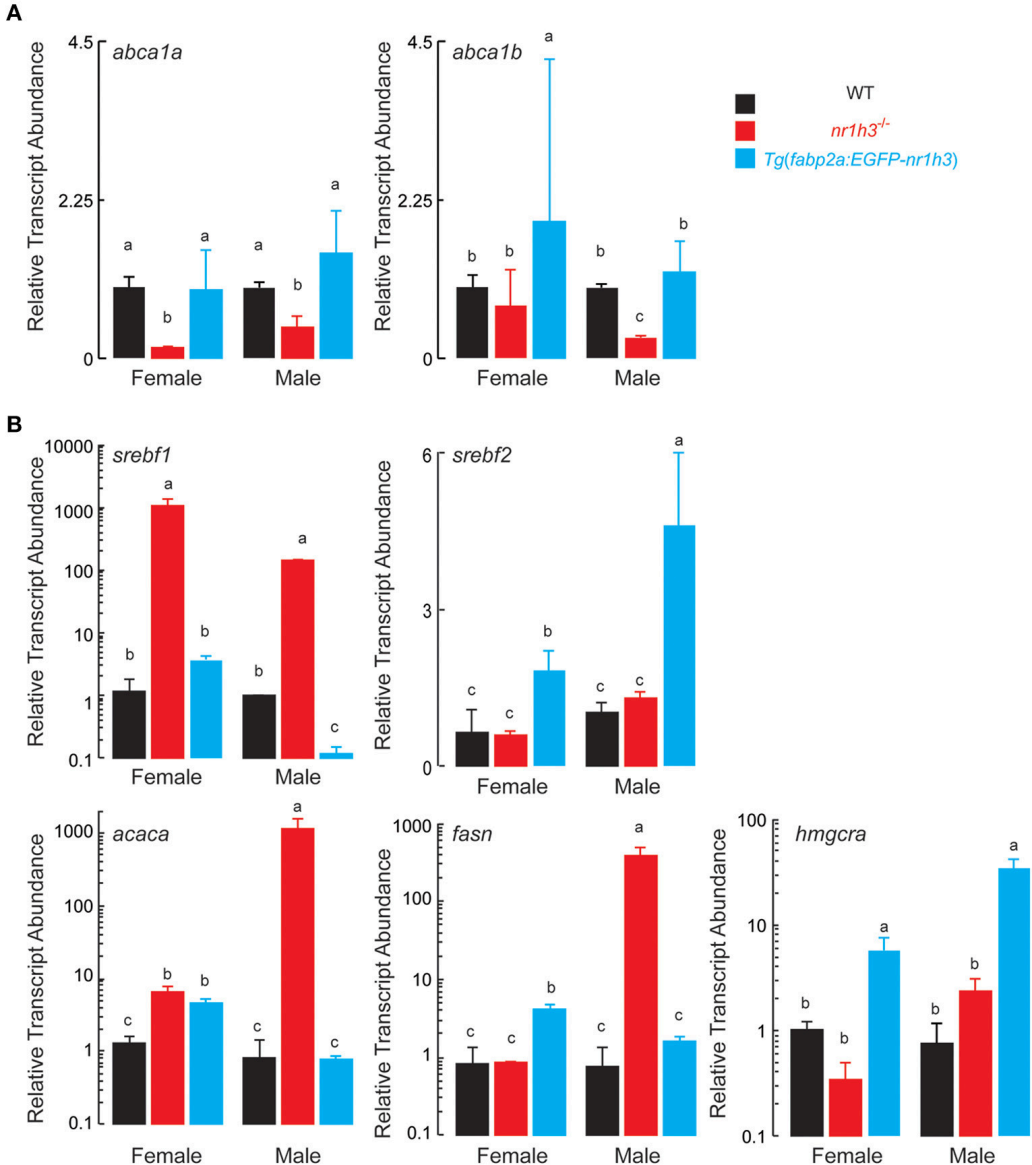
**Expression of Transcripts Encoding Components of Intestinal HDL Synthesis and Hepatic De Novo Lipogenesis. (A)** Livers were dissected from adult animals (*n* = 3 per sex) and *srebf1, srebf2, fasn*, and *acaca* transcripts were measured. Values not sharing a common superscript letter are significantly different at *P* < 0.05. **(B)** Intestines were dissected from adult animals (*n* = 3 per sex) fed the high cholesterol diets. RNA was extracted and *abca1a* and *abca1b* transcripts were quantified. Values not sharing a common superscript letter are significantly different at *P* < 0.05 in 1-way ANOVA.

Next, we measured the abundance of *abca1a* and *abca1b* transcripts in intestines to assess the effect of intestinal Lxrα over-expression on the expression of the basolateral sterol exporter that loads cholesterol onto HDL (Repa et al., 2000; Murthy et al., 2002). As expected, intestines *nr1h3*^−/−^ animals had lower expression of both *abc1a* and *abc1b*. *Tg*(*fabp2a:EGFP-nr1h3*) intestines from females showed increased *abc1b* abundance (Figure 5A).

Finally, because hepatic Lxrα is critical for cholesterol elimination, and it drives de novo lipogenesis (Zhang et al., 2012), we were curious to examine the effects of intestinal Lxrα over-expression on hepatic gene expression of *srebf1, srebf2* encoding the master transcriptional regulators of de novo lipogenesis and cholesterol biosynthesis, respectively, and representative target genes, *acaca, fasn*, and *hmgcra*. Critically, *srebf1* is a key direct target of Lxrα (Schultz et al., 2000; Rong et al., 2017). Figure 5B shows that *srebf1* and its two targets *acaca* and *fasn* were induced in *nr1h3*^−/−^ livers; while *srebf2* and its target gene *hmgcra* were induced in *Tg*(*fabp2a:EGFP-nr1h3*) livers.

## Discussion

The intestine can serve as a reservoir for absorbed dietary lipids in metazoans ranging from insects to fish, rodents, and humans (Robertson et al., 2003; Sieber and Thummel, 2009; Zhu et al., 2009; Douglass et al., 2012). The molecular signals controlling this storage function are beginning to emerge. We previously demonstrated that zebrafish Lxrα cell-autonomously regulates intestinal handling of absorbed lipids, with over-expression of Lxrα (i.e., increased activation relying on endogenous ligands only) diverting absorbed lipids to an enterocyte cytoplasmic storage pool by inducing expression of the long-chain acyl-CoA ligase gene *acsl3*, which encodes a lipid droplet-targeted enzyme that funnels CoA thioesters of fatty acids to neutral and phospholipids (Cruz-Garcia and Schlegel, 2014).

Here we developed novel methods for live imaging of larvae and biochemical assessment in adults to monitor lipid transport in zebrafish. This work was motivated by our observation that whole mount ORO staining was not sufficiently sensitive to discern differences between WT and *nr1h3*^−/−^ animals. In larvae we found using a more sensitive live imaging approach that intestinal over-expression of Lxrα delays and deletion of Lxrα increases the rate of transport of orally gavaged fluorescent lipid tracers. This relatively facile approach in larvae should be of use in future genetic and pharmacological studies to explore not only the consequences of modulating Lxr target gene dose, but in studying the effects of drugs on intestinal lipid handling. Since there is modest larval manipulation and serial measurement required (i.e., gavage, and repeated live imaging, with periods of free swimming at the euthermal temperature and normal light cycle conditions), a preclinical validation platform could emerge from the use of our methods. A moderate-scale chemical screen could also be based on this workflow with zebrafish larvae (Clifton et al., 2010).

In adults, we observed a sexually dimorphic role for Lxrα in regulating the rate of transport of absorbed lipids. Specifically, we found that genetic activation of intestinal Lxrα delayed the appearance of lipids in the circulation acutely in males. Differences in intestinal sterol transporters do not appear to account for these differences. Rather, there appears to be sexually dimorphic, intestinal Lxrα-directed differences in plasma Lpl activity. The molecular cues regulating the higher Lpl activity and relatively blunted post-prandial lipemic excursion in female zebrafish will require further study; however, it is important to note that women also have higher Lpl activity than men (Mittendorfer et al., 2003). This relative protection from post-prandial hyperlipidemia is reminiscent of the human epidemiological observations of relative cardiac protection in premenopausal women (Lozano et al., 2012). It is also reminiscent of the long-standing observation that 17-β-estradiol protects ovariectomized female mice from atherosclerotic progression in *Apoe*^−/−^ mice (Bourassa et al., 1996). Restoration of this physiological estrogen with subcutaneous pellets reduces total plasma cholesterol, VLDL/IDL cholesterol, and TAGs (Bourassa et al., 1996). Remarkably, 17-β-estradiol protects *Ldlr*^−/−^ mice from atherosclerosis; however, the effect does not appear to be related to changes in circulating lipoproteins, as measured in the non-fasting state (Marsh et al., 1999). Thus, there appear to be context-dependent atheroprotective roles for 17-β-estradiol in mice. In future studies, we will determine whether the sexually dimorphic post-prandial lipid excursion in zebrafish impacts atherogenesis. In both sexes, intestinal over-expression of Lxrα in the intestine blunted the development of hepatic cholesterol accumulation during a long HCD challenge, a feeding paradigm in which Lxrα deletion caused severe hypercholesterolemia in both sexes. Since our experimental approach fully suppressed Lpl activity in females and males, there are probably additional sexual modifiers of postprandial lipid excursion that merit additional study.

More generally, our results in a zebrafish model of Lxrα deletion and cell type-limited over-expression are important because postprandial dyslipidemia is not amenable to most currently available lipid-lowering therapies (Nordestgaard and Varbo, 2014). Our results suggest that selective activation of Lxrα in the intestine might serve to treat this condition. The atherosclerosis that emerges from repeated, prolonged bouts of exposure to TAG-rich lipoprotein particles in the post-prandial state might be ameliorated by intestine-limited Lxrα activation. Similar to HDCA, cholane and cholestane Lxr agonists might be useful for this purpose: while such ligands induce transcription of the master transcription factor driving de novo lipogenesis Srebf1, they simultaneously block Srebf1 proteolytic maturation by stabilizing the precursor in the endoplasmic reticulum, thereby avoiding the induction of hypertriglyceridemia and hepatic steatosis (Kaneko et al., 2003; Quinet et al., 2004; Peng et al., 2008, 2011; Kratzer et al., 2009). Non-sterol ligands lack the biophysical properties to arrest Srebf1 maturation, and their administration is marked by hepatic steatosis and hypertriglyceridemia (Schultz et al., 2000; Grefhorst et al., 2002; Bradley et al., 2007; Kirchgessner et al., 2016). Full dissection of the transcriptional program controlled by intestinal Lxrα, as well as dissection the reasons for the sexually dimorphic traits seen in our study, will provide a thorough mechanistic basis for developing new therapies that leverage the capacity of the intestine to store (and safely oxidize) absorbed fatty acids, while promoting net cholesterol elimination.

Finally, we note that the severe hypercholesterolemia seen in the *nr1h3*^−/−^ mutant animals fed the HCD provides a unique system for studying atherosclerosis: this degree of non-HDL cholesterol increase was achieved without additional genetic manipulations, reflecting conservation of aspects of lipoprotein metabolism other established models lack (Yin et al., 2012), such as the retention of an ortholog of the human Cholesteryl Ester Transfer Protein gene (Schlegel, 2016). We anticipate the live-imagine and adult physiology methods described here will allow the zebrafish model to be useful for preclinical testing of a range of lipid-lowering therapies.

## Author contributions

TB and AS designed the study, analyzed the data and wrote the paper. TB and SH performed experiments. All authors discussed the results and commented on the manuscript.

## Funding

This work was supported by a Grant In Aid from the Western States Affiliate of the American Heart Association to AS. (15GRNT24670009).

### Conflict of interest statement

The authors declare that the research was conducted in the absence of any commercial or financial relationships that could be construed as a potential conflict of interest.
